# Use of plasma nitriding to improve the wear and corrosion resistance of 18Ni-300 maraging steel manufactured by selective laser melting

**DOI:** 10.1038/s41598-021-82572-y

**Published:** 2021-02-08

**Authors:** M. Godec, B. Podgornik, A. Kocijan, Č. Donik, D. A. Skobir Balantič

**Affiliations:** Institute of Metals and Technology, Lepi pot 11, 1000 Ljubljana, Slovenia

**Keywords:** Engineering, Materials science

## Abstract

18Ni-300 maraging steel manufactured by selective laser melting was plasma nitrided to improve its wear and corrosion resistance. The effects of a prior solution treatment, aging and the combination of both on the microstructure and the properties after nitriding were investigated. The results were compared with conventionally produced 18Ni-300 counterparts subjected to the same heat- and thermo-chemical treatments. The plasma nitriding was performed under the same conditions (temperature of 520 °C and time of 6 h) as the aging in order to investigate whether the nitriding and the aging could be carried out simultaneously in a single step. The aim of this work was to provide a better understanding of the morphology and chemical composition of the nitrided layer in the additive-manufactured maraging steel as a function of the prior heat treatments and to compare the wear and corrosion resistance with those of conventional maraging steel. The results show that nitriding without any prior aging leads to cracks in the compound layer, while nitriding of the prior-heat-treated additive-manufactured maraging steel leads to benefits from the thermochemical treatment in terms of wear and corrosion resistance. Some explanations for the origins of the cracks and pores in the nitride layers are provided.

## Introduction

Selective laser melting (SLM) is one of the most promising additive-manufacturing (AM) technologies^1–3^. It is a powder-bed fusion process, where a high-power laser beam is used to selectively melt the powder feedstock in a layer-by-layer fashion^4^. A variety of metallic powders have been successfully employed, including pure metals (Ti, Al)^5–8^, alloys (steels, Ni-based alloys)^9,10^ and metal-matrix composites (MMCs)^11^. Compared with conventional manufacturing procedures, SLM offers several advantages, such as near-net-shape fabrication, the rapid production of components with complex geometries, little material waste and all this at an acceptable cost. For these reasons it has been widely applied in several strategic industries, including aerospace, automotive, biomedical and electronics as well as in tool manufacturing for the production of inserts with conformal cooling channels and components with high geometrical complexity^12,13^, i.e., the primary application fields of maraging steels^14,15^. Maraging steels, which belong to the group of tool steels, can be due to their low carbon content easily welded and also selective laser melted, and are therefore among the most used and researched in the field of AM technologies. The most investigated maraging steel for AM is the US classification 18Ni-300 (European, 1.2709; German X3NiCoMoTi 18-9-5)^4,13,16–18^.

In general, 18Ni-300 maraging steels belong to the class of low-carbon iron-nickel alloys with an exceptional strength-to-toughness ratio, good ductility, machinability and weldability, making them appropriate for use in aircraft, aerospace, moulds and tooling^12,19,20^. The ultra-high strength is the result of the hardening mechanism during the aging treatment, where the precipitation of nanometre-sized intermetallic particles (Ni_3_(Mo, Ti), Fe_2_Mo) takes place^21^. These particles are homogenously distributed in the low-carbon martensitic microstructure, where they inhibit the mobility of the dislocations and therefore enhance the microhardness and the strength. The high toughness and ductility originate from the ductile and machinable martensitic matrix^22,23^. However, compared to other tool steels or stainless steels, maraging steels exhibit some disadvantages, mainly related to their poor wear and corrosion resistance^20,24^. Surface-hardening processes such as plasma nitriding have been shown to be beneficial for improving the surface properties of conventionally produced maraging steel. The modified surface is provided by an insignificant reduction in the properties of the base material and with no dimensional change of the component^19,20,25^. Due to the very similar processing parameters (temperatures between 450 and 520 °C and times of 1–4 h) of the plasma nitriding process and the ageing cycle, it is possible to combine both processes into a single unique step. From the industrial point of view, this represents a significant advantage in terms of reducing the costs and times required^26^.

SLM is a very complex manufacturing technology that can produce microstructures and properties that can be substantially different to those obtained using conventional techniques. Therefore, the SLM process parameters need to be accurately set and controlled in order to obtain the appropriate microstructure and properties of the manufactured parts. Many investigations of additive-manufactured 18Ni-300 maraging steels dealing with the optimization of the process parameters^27,28^ and their influence on the mechanical properties and microstructure can be found in the literature^15,29–34^. There are only a few reports comparing AM with conventional maraging steel^31^ because it is difficult to compare those materials without considering the heat treatments. On the other hand, maraging steel still needs to be heat treated after the SLM process to achieve the desired properties. Further, it is to be expected that additional improvements for some applications in harsh environments can be achieved by plasma nitriding, as it is already well known for conventional maraging steel. The AM process leads to a cellular martensitic microstructure with nano-segregations and a small amount of retained austenite^31,35^. After the heat treatment the process of austenite reversion was reported^14^. Furthermore, the heat treatment for SLM maraging steel can also differ from traditional routes and needs to be optimised^36,37^. Therefore, a lot of attention has recently been devoted to post-process heat treatments, such as solution and aging treatments. Kempen et al.^29^ and Casati et al.^38^ studied the effects of different aging temperatures and times on the mechanical behaviour of SLM 18Ni-300 maraging steel. They found that a significant increase in the strength and a decrease in the ductility can be obtained after aging. Casati et al.^38^ also concluded that the solution treatment is not necessary and the as-built 18-Ni 300 maraging steel can be directly aged. Tan et al.^13^, on the other hand, showed that a combination of solution treatment and aging was crucial for an improvement of the mechanical performance. Yin et al.^39^ investigated the influence of aging temperature and aging time on the microstructure, mechanical properties (hardness, strength, ductility) and wear resistance. The maximum strength and wear resistance were obtained after aging at 490 °C for 3 h. Bai et al.^40^ performed a series of heat-treatment experiments, including solution treatment (ST), direct aging treatment (DAT) and a combined solution and aging treatment (SAT) on SLM maraging steel for the systematic investigation of their influence on the microstructure evolution, microhardness, tensile properties and toughness. The results show that similar mechanical properties can be achieved either by DAT or SAT. However, in terms of the practical application of SLM-processed maraging-steel parts, especially in the tooling industry, the lack of wear and the corrosion resistance still represent a significant challenge.

It is well known that nitriding can substantially increase the life span of conventionally processed tool inserts from maraging steel^41,42^. The positive effect of nitriding has also been reported for AM stainless steel 316L^43^. However, to the best of our knowledge, there are no reports in the literature on the plasma nitriding of AM maraging steel and its influence on the wear and corrosion properties. Therefore, the aim of this study was to investigate the effect of plasma nitriding combined with prior DAT and SAT conditions on the wear and corrosion resistance of SLM 18Ni-300 maraging steel. Based on a literature review, optimal SLM processing as well as post-heat-treatment conditions were applied. The results were compared to the conventional counterparts subjected to the same heat-treatment and nitriding process parameters. Although it would be of great interest to explain the influence of nitriding on the formation of the precipitates, the focus of this work was on an investigation of the influence of the heat-treatment conditions on the nitriding efficiency, the effect of nitriding on the wear and corrosion resistance, as well as a comparison of additive-manufactured and conventional maraging steel when subjected to nitriding. One of the important targets of the investigation was a comparison of the properties (microstructure, hardness, wear and corrosion resistance) of the AM as-built material with the conventionally manufactured material. The investigation of the precipitation behaviour and its correlation with the nitriding will be the subject of a separate publication.

## Results and discussion

**Figure 1:**
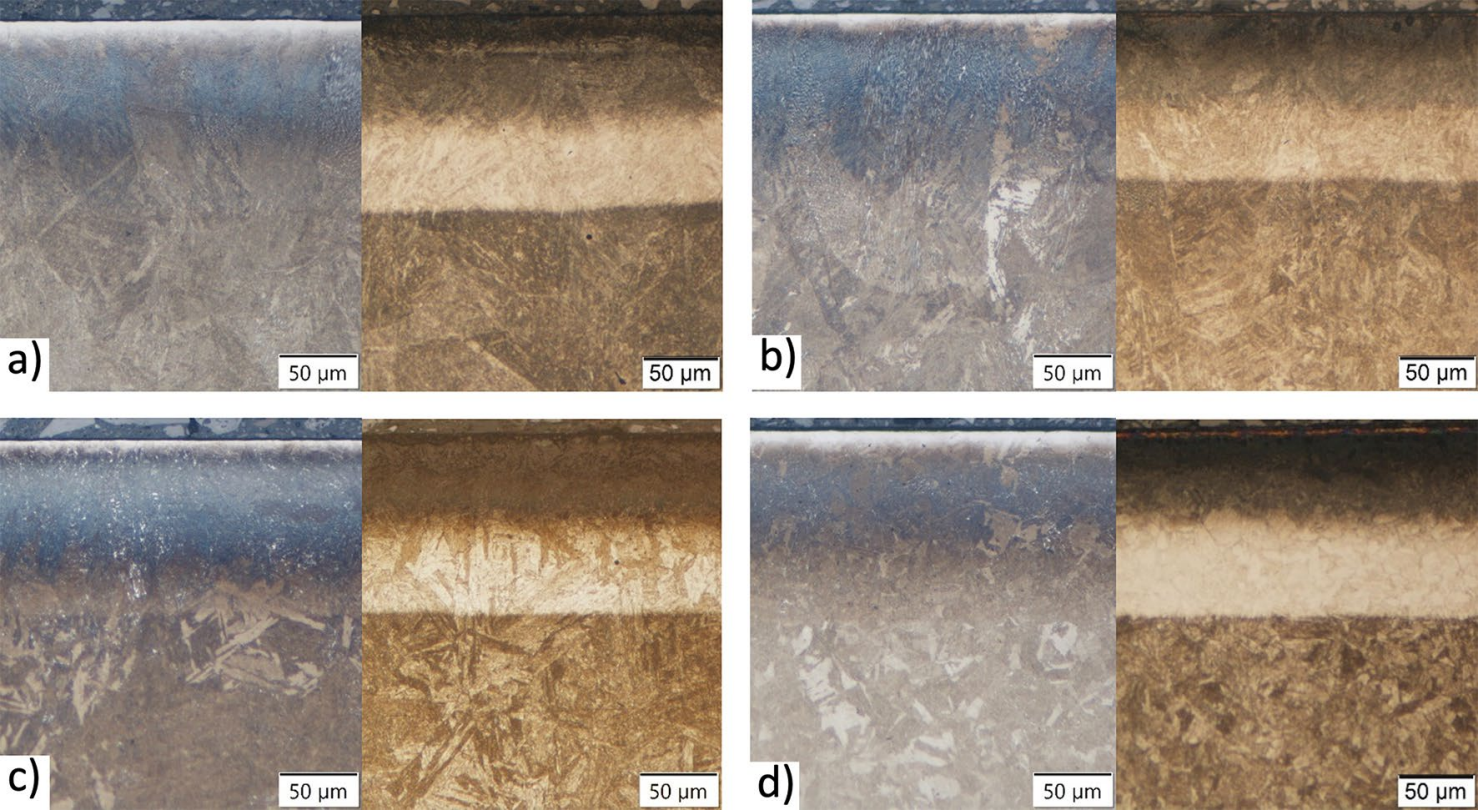
Light micrographs of a nitride layer etched by Nital (left part of the images) and ferric chloride (right part of the images) for maraging samples: (**a**) AM + N, (**b**) AM + DAT + N, (**c**) AM + SAT + N, (**d**) CM + SAT + N.

**Figure 2:**
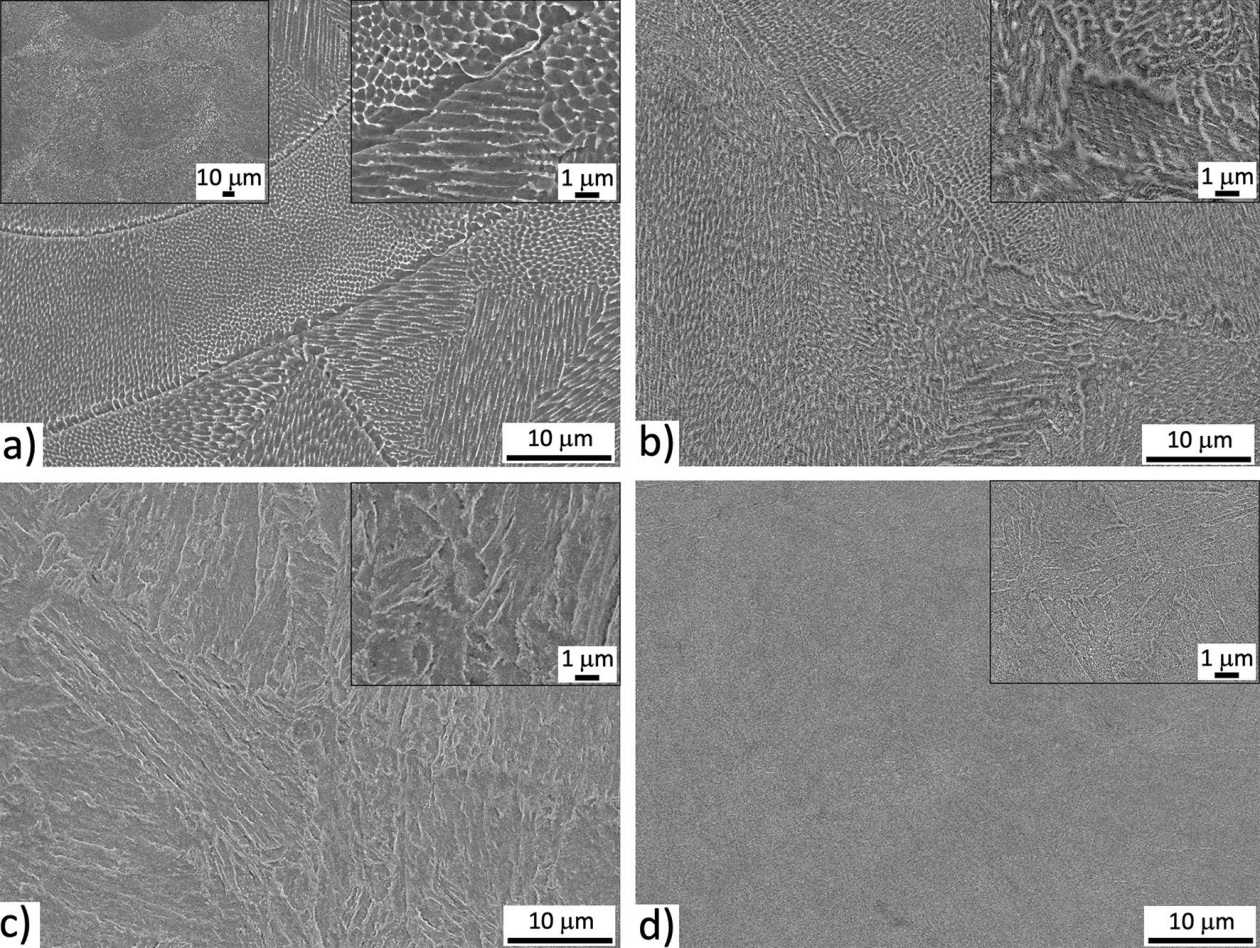
SE images of bulk microstructures of: (**a**) AM, (**b**) AM + DAT, (**c**) AM + SAT and (**d**) CM + SAT samples with insets of higher magnification; etched in Nital.

18Ni-300 maraging steel manufactured using an industrial SLM device was plasma nitrided in combination with and without a prior heat treatment to investigate their interacting influence on the wear and corrosion resistance. For a better understanding of the microstructure evolution and the formation of the nitride layer, the conventionally produced counterparts were thermochemically treated under the same conditions and compared with the AM samples. Due to the rapid solidification, the microstructure of the SLM as-built maraging steel differed significantly from the conventional steel and has a cellular/dendritic solidification microstructure^4,17^. Light micrographs (Fig. 1) present microstructures of the nitride layer on the as-built sample AM + N, differently thermochemically treated samples AM + DAT + N, AM + SAT + N and a conventional sample treated under the same conditions CM + SAT + N. The conventional maraging steel in the as-delivered state was solution treated; therefore, it does not make sense to study CM + DAT because it is identical to CM + SAT. The samples were etched in two different etchants (Nital left part of images, ferric chloride right part of images) to reveal all the details in the microstructure. The microstructure observed in the nitride layer as well as in the bulk is martensitic in all the samples, although in the CM sample the prior austenite grains that transform to martensite are significantly different. The prior austenite grains of the CM sample are polygonal, while in the AM samples the prior austenite grains have a more irregular shape due to the rapid solidification, which results in the different martensitic microstructure. Etching with Nital shows the melt pools and the morphology inside them, especially the cellular structure, which is better seen at higher magnifications and in detail with the SEM micrographs (Fig. 2). Melt pools can be observed in the as-built AM sample (Fig. 1a) as well as in the aged AM sample (Fig. 1b), with the melt-pool boundary running across the grains and having a slightly different chemical composition^44^. However, after the solution treatment the melt pools and boundaries disappear due to the diffusion process. The 8–10-µm-thick upper layer is not sensitive to the Nital etchant and most probably corresponds to the Fe_4_N compound layer^45^. The ferric chloride clearly reveals the nitride-layer thickness and also the grain structure in this layer, but only in the solution-treated AM sample (Fig. 1c) and the conventional reference sample (Fig. 1d). The depth of the nitride zone is very similar for all the samples and is between 115 and 130 µm. Visually, the nitride zone resembles the diffusion-layer structure with two regions of approximately equal thickness, with a different sensitivity to the ferric chloride etching (upper dark and bottom light), most probably due to the edge effect of the etching and the maximum residual stresses in the surface. The transition from the nitride layer to the bulk is smooth and visually restricted to a narrow range.

The bulk microstructure was investigated by SEM, where the melt pools are clearly visible and are 100–150-µm wide and 50–90-µm high (left inset in Fig. 2a). In Fig. 2a the cellular microstructure is visible and present in the whole volume. The dendrite cellular structure is a consequence of the rapid solidification and nano-segregation and is well described in the literature^31,46^. A higher-magnification image (right inset in Fig. 2a) shows very small (~ 100 nm) precipitates forming at the triple-cell junction during the AM process. The precipitates after aging (DAT) form within the whole volume; however, the growth is faster at the prior cell boundaries with a higher density of dislocations^47^. These features give the impression that the cell structure is still present after aging (Fig. 2b). After the solution treatment and aging (SAT) the cellular structure completely disappears, revealing the morphology of a martensite structure with fine precipitates (Fig. 2c). As the AM + SAT and CM + SAT undergo identical heat treatments, an identical microstructure is also expected. However, from the micrographs shown in Fig. 2c,d, coarser martensite laths can be seen for the AM sample. Furthermore, no precipitates are formed along the grain boundaries of the prior austenite grains and therefore these boundaries are almost invisible. On the other hand, in the CM sample the prior austenite grains are clearly visible with very fine precipitates located along the boundaries. The main reason for the coarser microstructure in the AM sample is the nature of the AM process, where elongated columnar grains are created. Therefore, the size of the grains defines the size of the martensite laths, which form as martensite packages inside those grains. The finer the grains, the finer are the packages.

**Figure 3:**
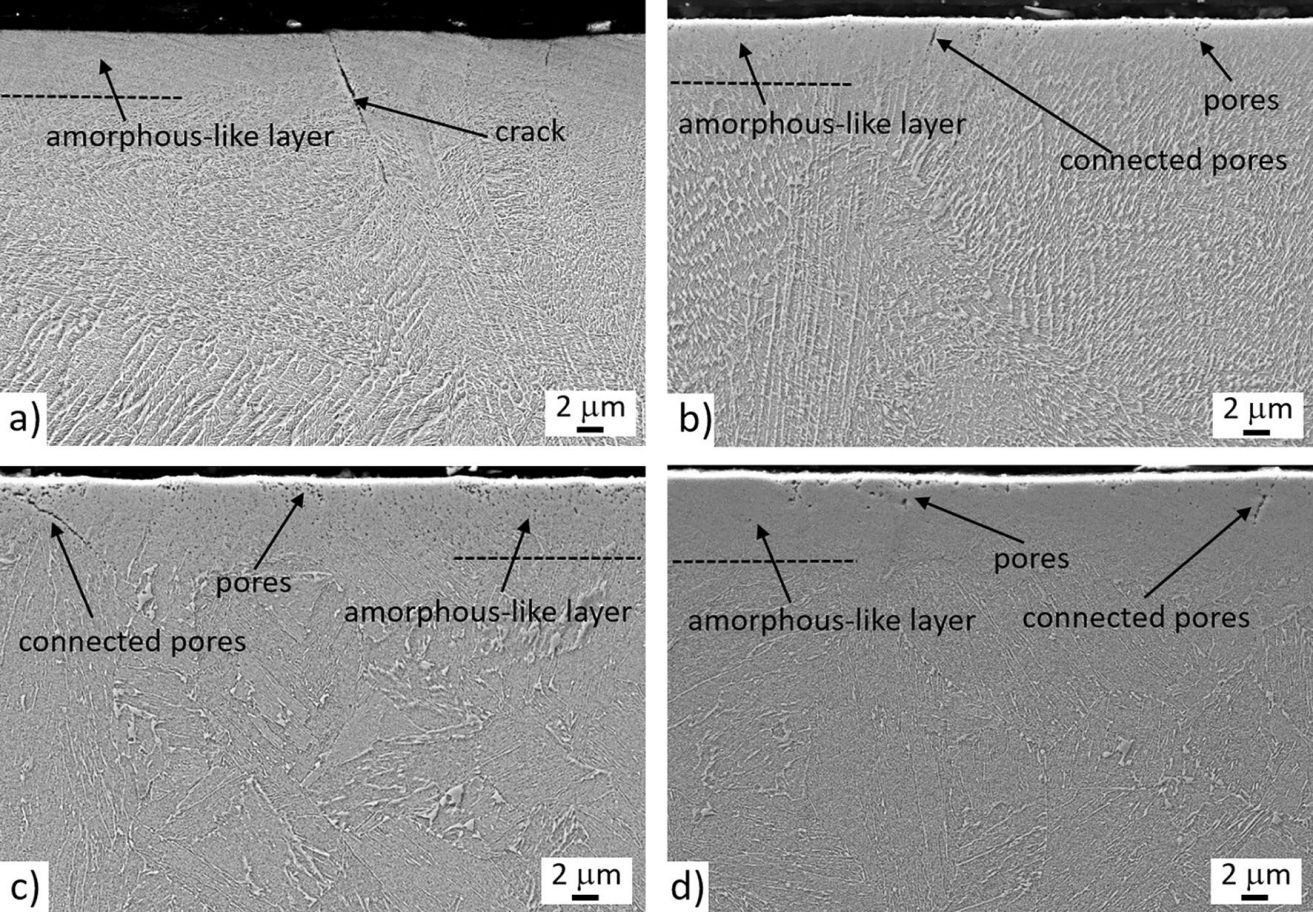
SE images of nitride layer for the investigated samples: (**a**) AM + N, (**b**) AM + DAT + N, (**c**) AM + SAT + N, (**d**) CM + SAT + N; etched in Nital.

Figure 3 presents the SEM image of the upper part of the nitrided zone (~ 30 µm). The thickness of the top, amorphous-like layer is approximately 5–10 µm, although it seems to be slightly thicker in the CM + DAT + N sample and most probably corresponding to the compound layer, which is very hard and prone to cracks. Higher magnification images of top most nitride layer can be found in the Supplementary Information with the appropriate Fig. S1. In the sample AM + N (Fig. 3a) small cracks are present in the compound layer, which can be assigned to the high concentration of internal stresses in the as-built AM material^48,49^. After aging no cracks are visible in the AM + DAT + N sample due to the stress release. However, some small pores (100–200 nm) can be observed in the surface and near-surface zones (up to a depth of 5 µm). Among the pores, some connected pores along the grain boundaries are also observed (Fig. 3b). The previously explained cellular morphology in the AM + N and AM + DAT + N samples is still visible. The solution treatment (SAT) removes the internal stresses and chemically homogenises the AM sample. The result is a disappearing of the cellular structure and the absence of cracks in the compound layer (Fig. 3c). The samples AM + SAT + N and CM + SAT + N have similar porosities in the top nitride layer, as well as connected pores (Fig. 3c and d). The transformation of the ε (Fe_2–3_N) phase to γ′ (Fe_4_N) causes an excess of nitrogen gas and forms the trapped pores beneath the surface^50,51^. The most reliable explanation for the absence of pores in the AM sample is the high dislocation density^44,46^, which allows a faster diffusion of the nitrogen.

**Figure 4:**
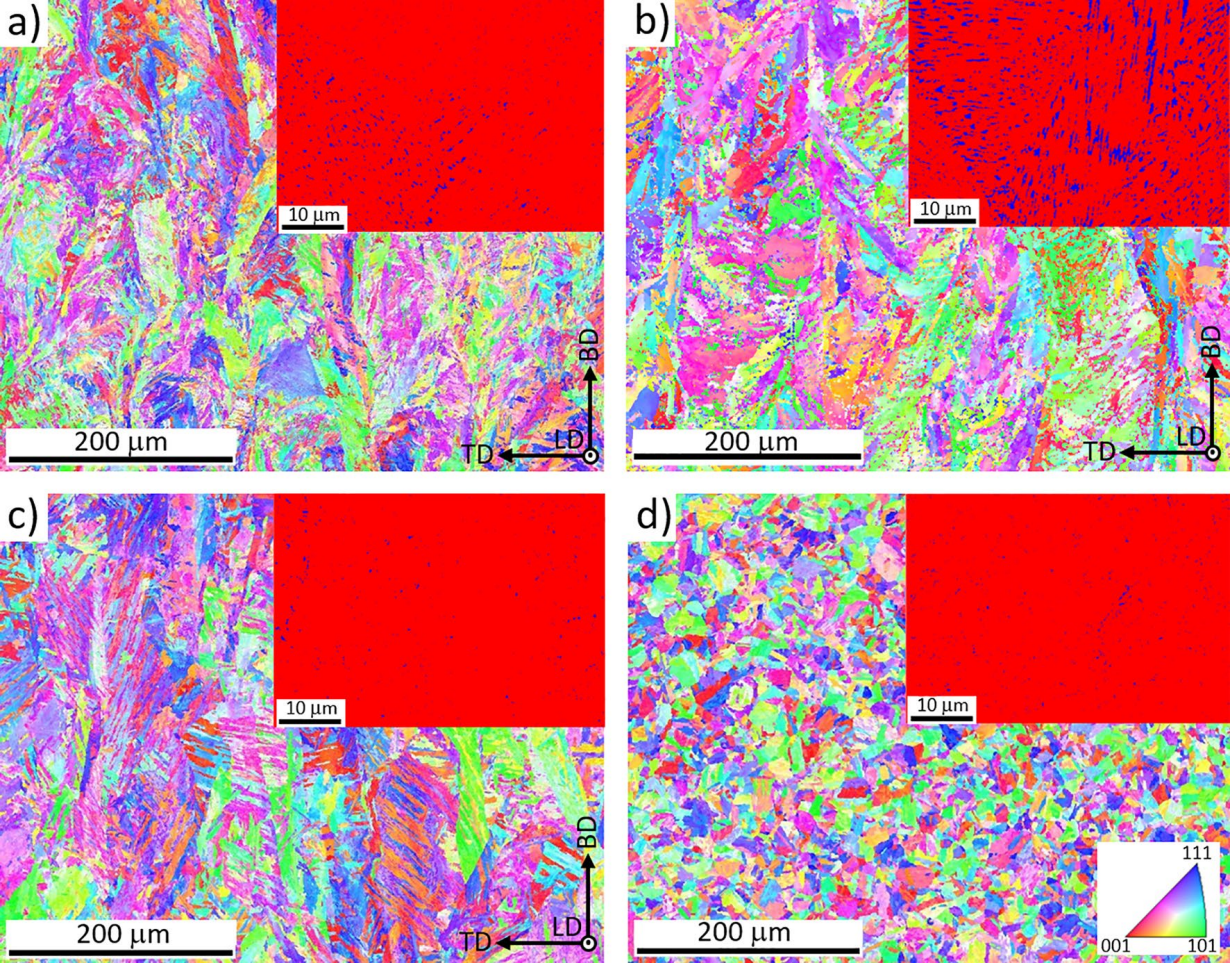
EBSD IPF maps in the BD direction with insets of EBSD phase analysis obtained at higher magnification of: (**a**) AM, (**b**) AM + DAT, (**c**) AM + SAT and (**d**) CM + SAT samples. LD, TD and BD stand for longitudinal, transverse and building direction, respectively.

The EBSD performed on the AM, AM + DAT, AM + SAT and CM + SAT samples indexed martensite with a small amount of austenite (retained/reverted) (Fig. 4), also reported by other authors^29,31,38^. The difference in the bulk microstructure between the AM and CM samples is the result of typical conditions during the SLM process, characterized by rapid solidification and multiple reheatings, which cause elongated grain growth in the building direction. The microstructure consists of lath martensite inside columnar grains (Fig. 4a–c). The heat-treated CM sample (CM + SAT) contains 1–2% of retained austenite and has small, polygonal prior austenite grains without an isotropic structure, which is typical for AM samples. The as-built AM sample, on the other hand, contains 3% of retained austenite. Aging increases the amount of austenite in the microstructure, since a larger amount of austenite is in equilibrium at the aging temperature, which remains in the microstructure after quenching and is known as reverted austenite^31^. Accordingly, the amount of austenite in the AM + DAT sample increased up to 11% (Fig. 4b). To avoid the austenite after aging a slower cooling rate should be used. However, when the AM sample is solution treated and aged (AM + SAT), again no more than 1–2% of austenite was detected (Fig. 4c). The significant difference in the amount of austenite between AM + DAT and AM + SAT can be explained by nano-segregations during the AM process, which cannot be eliminated only by aging. Therefore, a larger amount of Ni in certain areas, known as a γ-stabilizing alloying element, increases the formation of austenite. The absence of nano-segregations in the conventional sample is attributed to its chemically homogeneous structure. The selected SLM process parameters did not lead to any texture formation, as shown in Fig. 4. The previously described difference in the grain morphology, especially between the AM + SAT and CM + SAT, is even better seen in the EBSD IPF maps. The packages of martensite formed inside the large columnar grains in the AM material are much larger than the packages of martensite inside small polygonal grains in the CM material (Fig. 4d).

**Figure 5:**
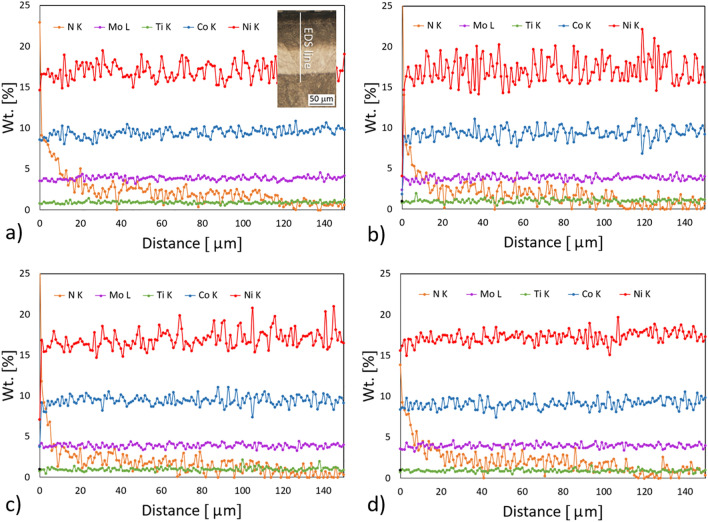
EDS line-scan analyses of: (**a**) AM, (**b**) AM + DAT, (**c**) AM + SAT and (**d**) CM + SAT samples.

**Figure 6:**
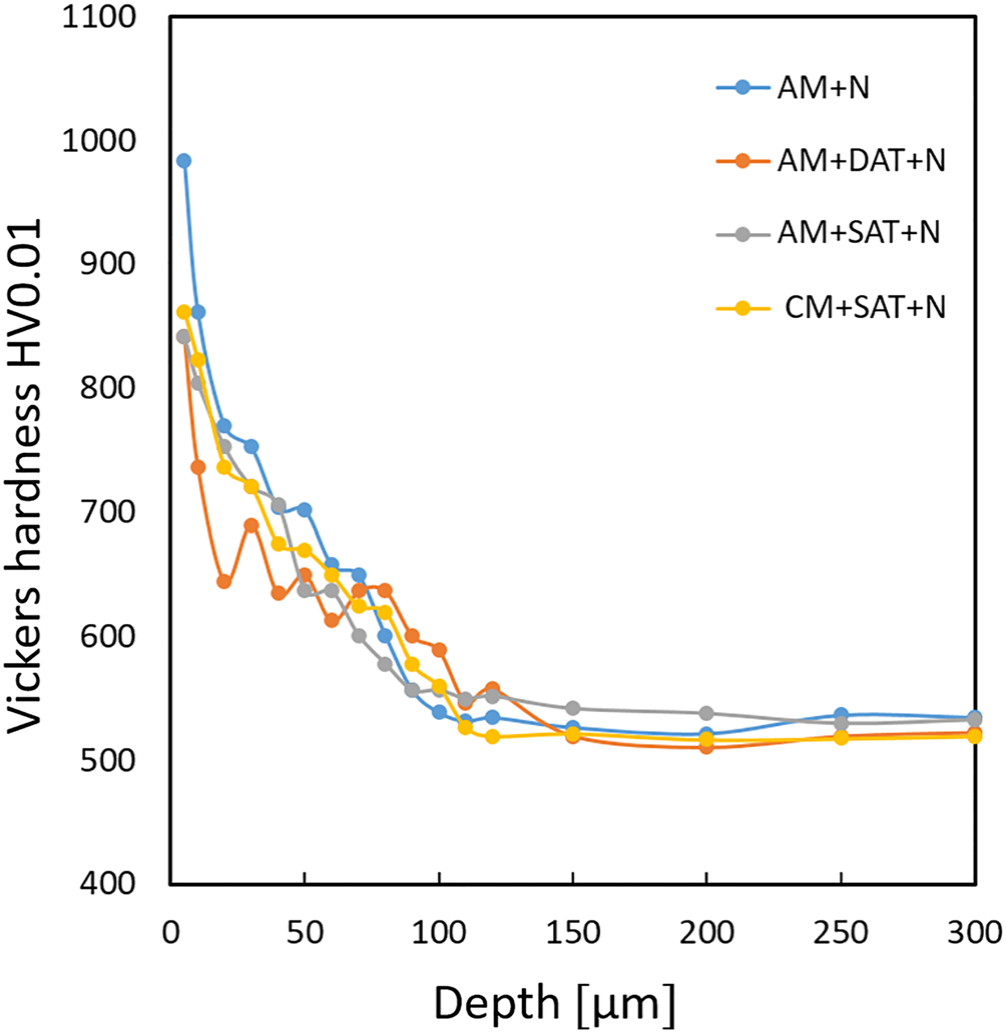
Microhardness depth profiles of: (**a**) AM + N, (**b**) AM + DAT + N, (**c**) AM + SAT + N and (**d**) CM + SAT + N samples.

The nitride layers were characterized using an EDS line-scan analysis to explain the elemental distribution, especially the depth of the nitrogen penetration. The EDS line analyses performed on the cross-section samples are shown in Fig. 5. The inset in Fig. 5a schematically shows the EDS line path through the nitride layer, which was similar for all four samples. In all the samples, the nitrogen signal correlates well with the depth of the nitride layer, which is 115–130 µm, as seen from the LM micrographs. The content of nitrogen starts to increase in the top 20 µm, which corresponds well with the compound layer visible in the SEM images (Fig. 3) and the hardness depth profile (Fig. 6). The Ti, Mo and Co values are similar and almost constant in all the samples. The signals for nickel show a high scatter, assigned to the redistribution of the nickel due to the higher solubility of the nickel in austenite. It is well known that Ni stabilises the austenite phase. The highest scatter is observed for the AM + DAT sample (Fig. 5b), which contains the largest amount of austenite. Although the AM samples contain more dislocations and other crystal-lattice defects^44^, no significant increase in the thickness of the diffusion layer is evident (Fig. 1), while the amount of nitrogen in the nitride layer is almost the same for all samples (Fig. 5). Based on EDS line analysis no significant effect of the heterogeneous structure on the nitriding kinetics is observed.

Bulk hardness measurements of the samples before nitriding show that the as-built sample (AM) has a lower hardness of 39 HRC (387 equivalent HV), compared to the heat-treated ones (AM + DAT, AM + SAT and CM + SAT) with a hardness of 49–50 HRC (500–515 equivalent HV). The AM sample shows the lowest hardness value because the precipitates do not form (or at least not completely) during the SLM process^14^. The sample AM + DAT contains the largest amount of austenite; therefore, a lower hardness value would be expected. However, the dislocation cellular structure is not completely removed during aging and this contributes to the higher hardness value, which in the end gives similar hardness values to all the heat-treated samples. The Vickers microhardness depth profiles of the nitrided layer for the AM + N, AM + DAT + N, AM + SAT + N and CM + SAT + N samples are shown in Fig. 6. The bulk hardness for all the nitrided samples is very similar, i.e., in the range of 520 HV0.01. However, slightly lower values as well as a larger scatter within the nitride layer can be observed for the AM + DAT sample, which is attributed to the largest amount of austenite phase in the microstructure. It also shows a more pronounced drop in the surface hardness, which agrees with the higher austenite content and the slightly thinner compound layer, as visible from the SEM cross-section micrographs (Fig. 3). In terms of the surface hardness of the nitride layer, the AM + N sample shows the highest hardness of the compound layer, around 1150 ± 35 HV0.01, while the hardness of the other three samples is about 850 ± 40 HV0.01. The as-built sample has no precipitates, which corresponds to the lowest bulk hardness value. However, during nitriding, both processes (the formation of precipitates and nitrides) take place and increases the hardness to the highest value for all the studied samples. The higher hardness is also due to the cellular structure, with its high dislocation density and the nano-oxide particles pinning the dislocations^46^. On the other hand, in all the samples the hardness decreases with the depth, reaching the value of the base material at a depth of approximately 120 µm, correlating well with the LM images (Fig. 1).

**Figure 7:**
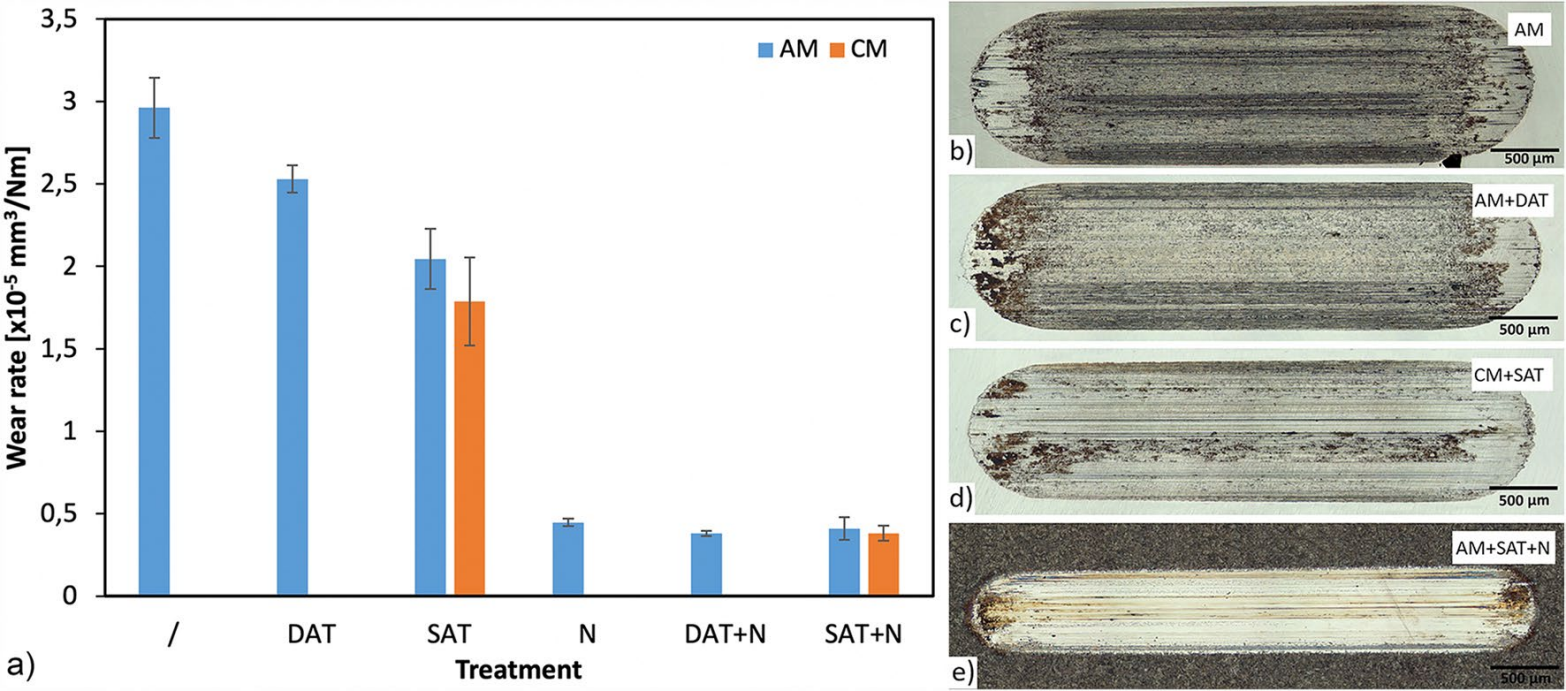
(**a**) Wear rates for the investigated AM and CM maraging-steel specimens and micrographs of the wear tracks of: (**b**) AM, (**c**) AM + DAT, (**d**) CM + SAT and (**e**) AM + SAT + N samples.

In terms of wear resistance (Fig. 7a), the highest wear rate (wear volume divided by normal load and total sliding distance) in the range of 3.0 × 10^–5^ mm^3^/Nm was obtained for the AM sample. This is due to the high internal stresses, nano-segregations and the small number of precipitates, resulting in a lower bulk hardness (40 HRC) and combined abrasive/adhesive wear, as shown in Fig. 7b. Although the sample is thermally exposed several times during the laser melting of its upper layers, it is just for very short periods of time, which cannot replace the post-aging procedure, which gives the maraging steel its final properties (i.e., high bulk hardness of 48–50 HRC) due to the precipitates’ formation (Ni_3_(Ti,Al). Interestingly, the aging treatment itself does not significantly improve the wear resistance of the AM sample (AM + DAT). The wear rate is reduced by less than 15%, with the adhesive wear component still being very strong (Fig. 7c). The main reason lies in the high retained-austenite content. A further improvement in the wear resistance of the AM samples, on the other hand, is obtained by a combination of solution and aging treatment (AM + SAT), providing a high hardness and a more homogeneous microstructure. This results in a reduced abrasive-wear component and an about 40% improvement in the wear resistance, as compared to the as-built AM sample. However, regardless of the heat-treatment conditions the AM samples with a reduced microstructure homogeneity show an about 15% lower wear resistance than the conventional samples treated under the same conditions and showing the same bulk hardness. Although the conventional maraging steel is delivered in the soft-annealed condition, the solution treatment additionally eliminates the macro-segregations, thus providing a further improvement in the wear resistance (Fig. 7d). An even more drastic increase in the wear resistance, obtained for all the samples, was provided by plasma nitriding, as shown in Fig. 7a. The wear rates were reduced by 4–7 times. The high hardness of the nitride layer eliminated the adhesive-wear component, at the same time providing a superior abrasive wear resistance (Fig. 7e). The formation of the hard nitride layer more-or-less eliminates the negative SLM effects as well as the heat-treatment history, with all the samples showing very similar wear rates of about 0.4 × ·10^–5^ mm^3^/Nm and the wear being concentrated within the top 10 µm of the nitride layer. However, for the AM material, the presence of microcracks in the nitride layer of the as-built sample (AM + N) and the significant number of pores in the near-surface area of the AM + SAT + N sample result in somewhat higher wear rates and more scatter (Fig. 7a). A detailed examination of the friction behaviour of the tested samples is explained in the Supplementary text with the appropriate Fig. S2 and Fig. S3.

Potentiodynamic polarization curves for all the AM and CM samples measured in 3.5% NaCl are presented as Supplementary Fig. S4. The corrosion potentials (*E*_corr_), corrosion current densities (*i*_corr_) and corrosion rates (*v*_corr_) are listed in Table 1. Based on the results of the corrosion measurements, the as-built AM sample has a similar corrosion resistance to the AM + DAT and AM + SAT samples. The as-built AM sample has a high dislocation density, internal stresses^49^ and chemical nano-segregations^31^. The internal stresses and chemical nano-segregations make a negative contribution to the corrosion properties, while the absence of precipitates has the opposite effect. The slightly increased corrosion of the AM + DAT and AM + SAT samples is probably the result of precipitate formation during the aging. The nitriding improves the corrosion resistance, due to the formation of a compound layer, which is known to be corrosion-resistant^52^. Our results show that the nitrided samples exhibit a passive region in the potentiodynamic curves, compared to the samples prior to nitriding, where this region was not observed (see Supplementary Fig. S4). In fact, nitriding without a prior heat treatment achieves more-or-less the same corrosion rate. However, without the prior heat treatment, due to the high internal stresses some cracking of the nitride layer is observed (Fig. 3a). In general, the corrosion rates for AM are higher than for the CM samples due to the appearance of local stresses caused by the multiple melt cavities, the dendritic cellular structure and the increased roughness, which results in increased corrosion in these areas^53^. A comparison between the AM + SAT and CM + SAT samples shows the improved corrosion resistance of the conventionally produced material due to its better chemical homogeneity, particularly since pores are not present in such material. In the SLM-produced materials, the number of pores is small, but they cannot be completely avoided, which results in a slightly lower corrosion resistance. The sample CM + SAT shows a similar corrosion resistance to the nitrided samples, which can be attributed to the fine-grained structure with fine martensite laths. This is also an explanation for the better corrosion properties of the CM + SAT sample compared to the AM + SAT sample, although in both cases the same heat treatment was performed.

**Table 1:** Electrochemical parameters determined from the potentiodynamic curves.

Material	*E*_corr_ (mV)	*i*_corr_ (µA/cm^2^)	*v*_corr_ (µm/year)
AM	− 319 ± 4	1.30 ± 0.05	15.0 ± 0.5
AM + DAT	− 327 ± 4	1.35 ± 0.03	15.5 ± 0.5
AM + SAT	− 231 ± 2	1.65 ± 0.04	18.9 ± 0.5
CM + SAT	− 349 ± 4	0.87 ± 0.01	10.0 ± 0.2
AM + N	− 314 ± 3	0.88 ± 0.02	10.0 ± 0.3
AM + DAT + N	− 271 ± 2	1.06 ± 0.03	12.2 ± 0.4
AM + SAT + N	− 307 ± 3	0.87 ± 0.02	9.9 ± 0.2
CM + SAT + N	− 256 ± 2	0.93 ± 0.03	10.7 ± 0.3

## Conclusions

In this study the influence of plasma nitriding in combination with different prior-heat-treatment processes on the wear and corrosion resistance of AM maraging steel was investigated. The results were compared with conventional maraging steel subjected to the same heat and thermo-chemical processes. The following conclusions can be drawn:

- As-built AM material contains a small amount of retained austenite because of nano-segregations caused by rapid solidification during the AM process. The aging treatment leads to more reverted austenite, while after the solution treatment and aging a larger amount of austenite is not obtained. After aging, the samples still have a lot of chemical inhomogeneity, which causes the formation of austenite. Therefore, a slow cooling rate is essential for a smaller amount of austenite. The microstructure of the AM material is similar in all the investigated conditions (as-built, directly aged, solution treated and aged) and consists of elongated columnar grains with martensite laths inside them. On the other hand, the microstructure of the conventional material is much finer in terms of the grains and martensite laths.
- Plasma nitriding was shown to be a beneficial thermo-chemical treatment for the AM maraging steel (18Ni-300) in terms of improving the wear and corrosion resistance. The nitride layer consists of the compound and diffusion layers with thicknesses of 5–10 µm and 100–120 µm, respectively. The thicknesses of the layers correlate very well with the nitrogen concentration in the surface. Plasma nitriding without prior heat treatment results in a small crack within the compound layer due to the high internal stresses, which diminish the surface resistance to wear. On the other hand, for the plasma nitriding on the heat-treated samples, very small pores of up to 200 nm were formed in the compound layer. Despite this, the AM and conventional samples, heat treated prior to nitriding, had the best and very similar wear resistances.
- Plasma nitriding has a positive effect on the corrosion resistance, which can be observed in the formation of a passive region. However, in general, AM-produced samples have higher corrosion rates than their conventional counterparts due to the greater inhomogeneity, some porosity and the appearance of local stresses caused by multiple melt cavities, a dendritic cellular structure and an increased roughness.
- For the conventional maraging steel, it was already shown that it is possible to combine the aging and nitriding into a single step. One of the aims of the present research was to confirm the same for AM materials. However, the results show that this is not the optimal solution for the AM material, due to crack formation in the compound layer. The nitriding with the prior heat treatment (i.e., aging or solution treatment and aging) leads to the formation of a crack-free nitride layer, required in the case of tool inserts subjected to heavy loads and severe contact conditions. On the other hand, the short annealing time for stress release might be sufficient to prevent cracking during the nitriding while maintaining a high dislocation density, which enables a pore-free compound layer.

## Materials and methods

### Materials

Gas-atomized powder feedstock (MS1) supplied by EOS, corresponding to maraging steel grade 18Ni-300 (C ≤ 0.003, Si ≤ 0.10, Mn 0.04, S ≤ 0.001, Co 8.8, Cr ≤ 0.1, Mo 5.0, Ni 17.8, Ti 0.80 wt%), was used for the SLM built parts. The size of the powder particles was less than 63 μm, with the majority in the range from 15 to 45 μm. SLM samples of 30 × 30 × 30 mm^3^ were built in an industrial AM machine (EOS EOSINT M280) with 400-W fibre laser, using commercial process parameters: laser power of 285 W, laser speed 960 mm/s, distance between the laser paths 0.11 mm, hatching in x and y, alternating and rotating.

Commercial 18Ni-300 maraging (C ≤ 0.002, Si ≤ 0.10, Mn ≤ 0.1, S ≤ 0.001, Co 8.5, Cr ≤ 0.1, Mo 4.9, Ni 17.8, Ti 0.68 wt%) steel in the as-delivered (soft-annealed) state was used as a conventionally manufactured counterpart. Chemical compositions were determined with an X-ray fluorescence spectrometer XRF (Thermo Scientific Niton XL3t GOLDD+) and ELTRA Elemental Analyzer CS800 (for carbon and sulphur).

### Heat and thermochemical treatment (plasma nitriding)

A post-process heat treatment is essential for both SLM and conventionally manufactured maraging steel in order to improve the mechanical properties. The heat treatment was performed in two different stages, with the parameters (temperature and time) chosen on the basis of the literature data^40^:

- Direct aging at 520 °C for 6 h (DAT)
- Solution treatment at 840 °C for 30 min + aging treatment at 520 °C for 6 h (SAT)

For the enhancement of the surface properties, such as wear and corrosion resistance, the samples were thermochemically treated by plasma nitriding. Plasma nitriding (N) was performed on the prior heat-treated samples (DAT or SAT) at the nitriding temperature of 520 °C. Due to the identical aging and nitriding temperature, the SLM sample was also nitrided in the as-built state (without any prior heat treatment) in order to investigate the possibility and effectiveness of joining the two processes in a single step.

A Metaplas Ionon HZIW 600/1000 cold wall reactor, equipped with an internal convection-heating system and an internal gas/water heat exchanger for rapid cooling, was used for the plasma nitriding. The convection and plasma heating of the specimens to the sputtering temperature of 450 °C and processing temperature took approximately 3.5 h. The soaking time at the nitriding temperature was 6 h in a gas mixture of 75 vol% H_2_: 25 vol% N_2_.

### Microstructure characterization

The microstructures were characterized on cross-sectioned metallographic samples [mounted in conductive Bakelite resin, grinded, finally polished using 1-μm diamond suspension and etched in Nital (2% Nitric Acid, Ethyl Alcohol) and ferric chloride (FeCl_3_)] using a light microscope (Nikon Microphot FXA) equipped with a digital camera (Olympus DP73).

A field-emission scanning electron microscope (ZEISS Cross Beam 550 FIBSEM) was used for the energy-dispersive spectroscopy (EDS) at 15 kV and 5 nA for mapping and 2 nA for line scanning analyses, as well as for secondary-electron (SE) imaging. Electron-backscatter diffraction (EBSD) was employed with a Hikari Super EBSD Camera at 70° with an accelerating voltage of 15 kV and a probe current of 10 nA to observe the microstructure with the included TEAM EDAX software, and for the data post processing, OIM software was employed.

### Wear testing and hardness measurements

A reciprocating sliding-wear test machine with the ball-on-disc contact geometry was employed to determine the wear behaviour (wear volume and coefficient of friction) of the samples. A 20-mm-diameter Al_2_O_3_ ball was used as the oscillating counter-body to simulate the abrasive wear mechanism and concentrate the wear on the steel-disc samples. Dry sliding-wear tests were performed at a normal load of 20 N, corresponding to a nominal contact pressure of 900 MPa, and an average sliding speed of 0.12 m/s (amplitude 4 mm and frequency 15 Hz). The overall testing time was 1000 s, resulting in a total sliding distance of 120 m. Three parallel tests at room temperature were performed on all the samples, which were mirror polished before the nitriding. After the test, the wear volume was measured directly by analysing the wear track with a high-resolution 3D optical microscope (Alicona InfiniteFocus G4 3D) based on a variation of the focus, intended for topography and form measurements. On the other hand, the coefficient of friction was measured continuously as a function of the testing time and sliding distance, with the average values obtained during the steady-state conditions that were calculated.

Depth profiles HV0.01 of the nitride layers were measured using an Instron Tukon 2100 B Vickers hardness tester. Bulk hardness before nitriding was measured using an Instron B2000 Rockwell hardness tester (HRC).

### Electrochemical measurements

The electrochemical evaluation was performed in a 3.5% NaCl solution at room temperature using a three-electrode system with the test specimen as the working electrode, a saturated calomel electrode (SCE, 0.242 V vs. SHE) as the reference electrode and a platinum mesh as the counter electrode. All the samples were stabilized at the open-circuit potential (OCP) for 1 h prior to the measurement. The potentiodynamic curves were measured at a scan rate of 1 mV/s on a Potentiostat/Galvanostat/FRA instrument (BioLogic SP-300 with EC-Lab V11.27 software). All the measurements were repeated three times to obtain statistically relevant results.

## Supplementary Information

Supplementary Information.
