# Supplementary material for: Use of plasma nitriding to improve the wear and corrosion resistance of 18Ni-300 maraging steel manufactured by selective laser melting
Source: Sci Rep. 2021 Feb 8;11:3277. doi: 10.1038/s41598-021-82572-y (PMC7870834; doi:10.1038/s41598-021-82572-y)
Supplement: Supplementary file 1 — Supplementary Information. [file 41598_2021_82572_MOESM1_ESM.docx]

**Supplementary information**

**Use of plasma nitriding to improve the wear and corrosion resistance of 18Ni-300 maraging steel manufactured by selective laser melting**

M. Godec*, B. Podgornik, A. Kocijan, Č. Donik, D. A. Skobir Balantič

Institute of Metals and Technology, Lepi pot 11, 1000 Ljubljana, Slovenia

*Corresponding author E-mail address: [matjaz.godec@imt.si](mailto:matjaz.godec@imt.si)

Magnified SE images of Fig. 3 showing upper part of nitride layer for the investigated samples are presented in **Fig. S1**. A typical coefficient-of-friction curve, obtained during the reciprocating abrasive wear testing of the maraging steel samples, is shown in **Fig. S2**. Similar behaviour with running-in (the first 50 seconds) and steady-state phase (100 to 1000 sec) was observed for all the investigated specimens. However, certain differences in the average values are evident, as shown in **Fig. S3**. For the AM samples the aging treatment (DAT) increases the steady-state friction (by about 10 %), making it more unstable during the second part of the testing due to the increased austenite content and precipitation. The combination of the solution treatment and aging (SAT), on the other hand, results in a reduced coefficient of friction, especially during the running-in phase, obtained by eliminating the nano-segregations, reducing the retained austenite content and providing a more homogeneous microstructure. Lower friction is provided by nitriding and the formation of a hard nitride layer, being in the range 0.6–0.7. However, micro-cracks found in the compound layer for the as-built specimen (AM+N) and the presence of pores and nano-segregations for the heat-treated specimens hinder the low-friction performance on a longer run. Compared to the conventionally produced maraging-steel specimens, the AM specimens show a similar friction after the heat treatment, being age hardening (DAT), having a combined solution treatment and age hardening (SAT) or nitriding. The higher initial friction for the solution treated and aged AM specimen compared to the conventional one can be related to the less homogeneous microstructure.


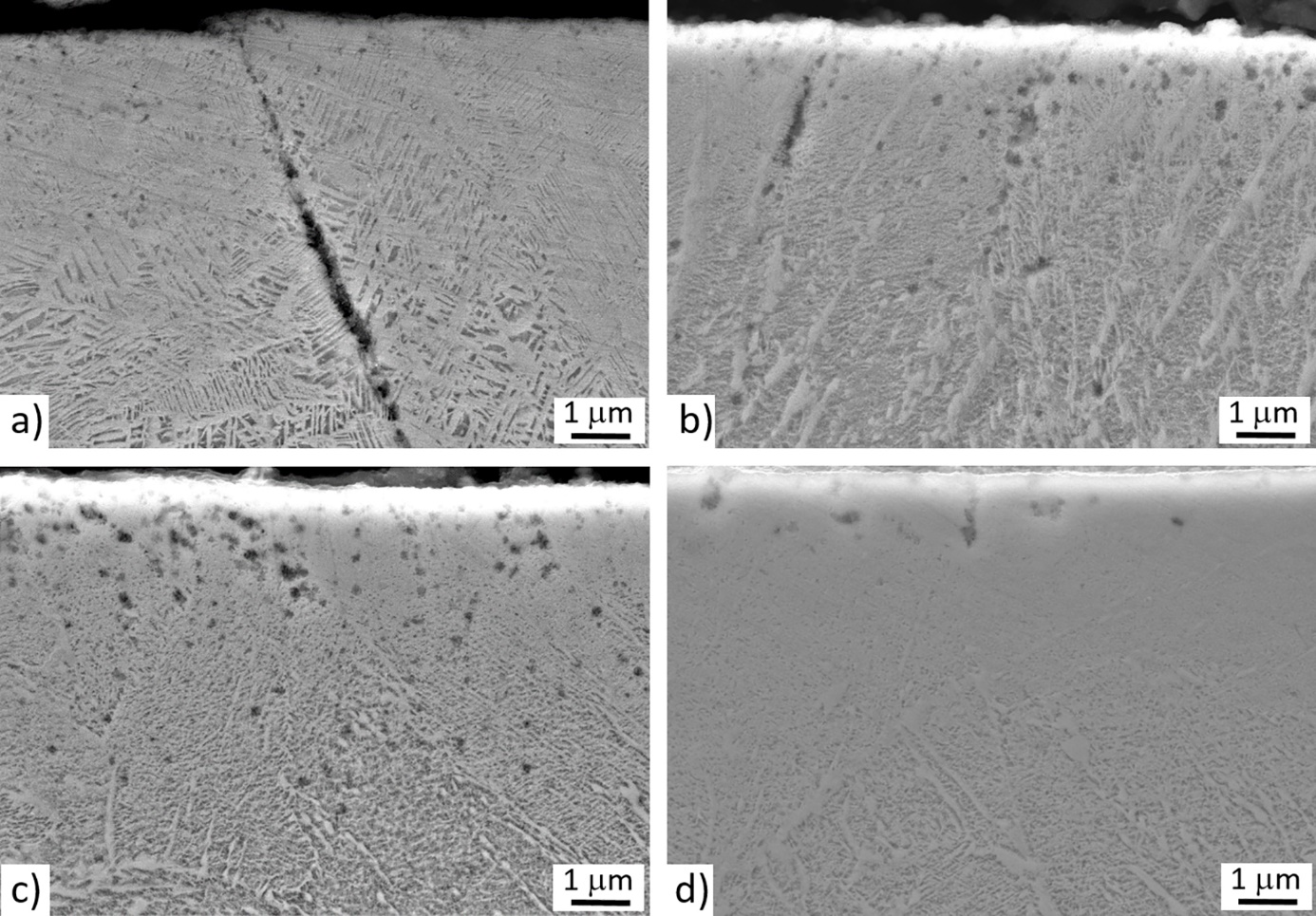


**Figure S1.** Magnified SE images of nitride layer for the investigated samples: (a) AM+N, (b) AM+DAT+N, (c) AM+SAT+N, (d) CM+SAT+N; etched in Nital.


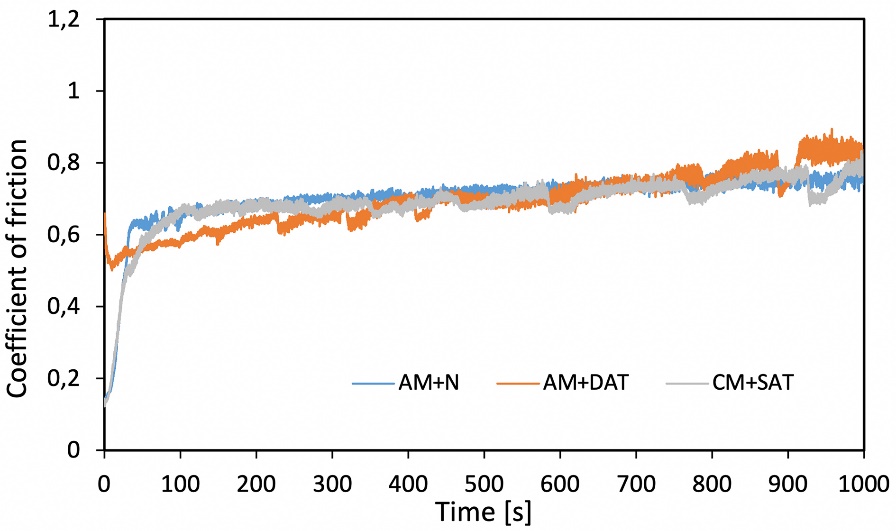


**Figure S2.** Typical coefficient of friction curve recorded during reciprocating dry sliding test.


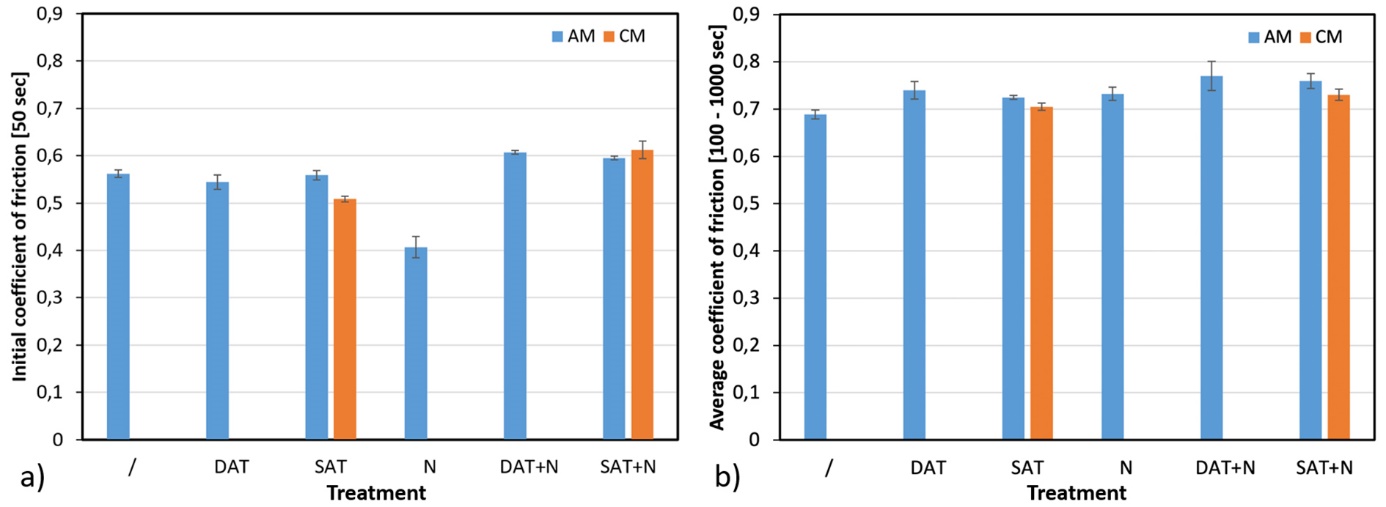


**Figure S3.** (a) Initial and (b) steady-state coefficient of friction for the investigated maraging-steel specimens (AM – additive manufactured, CM – conventionally produced).


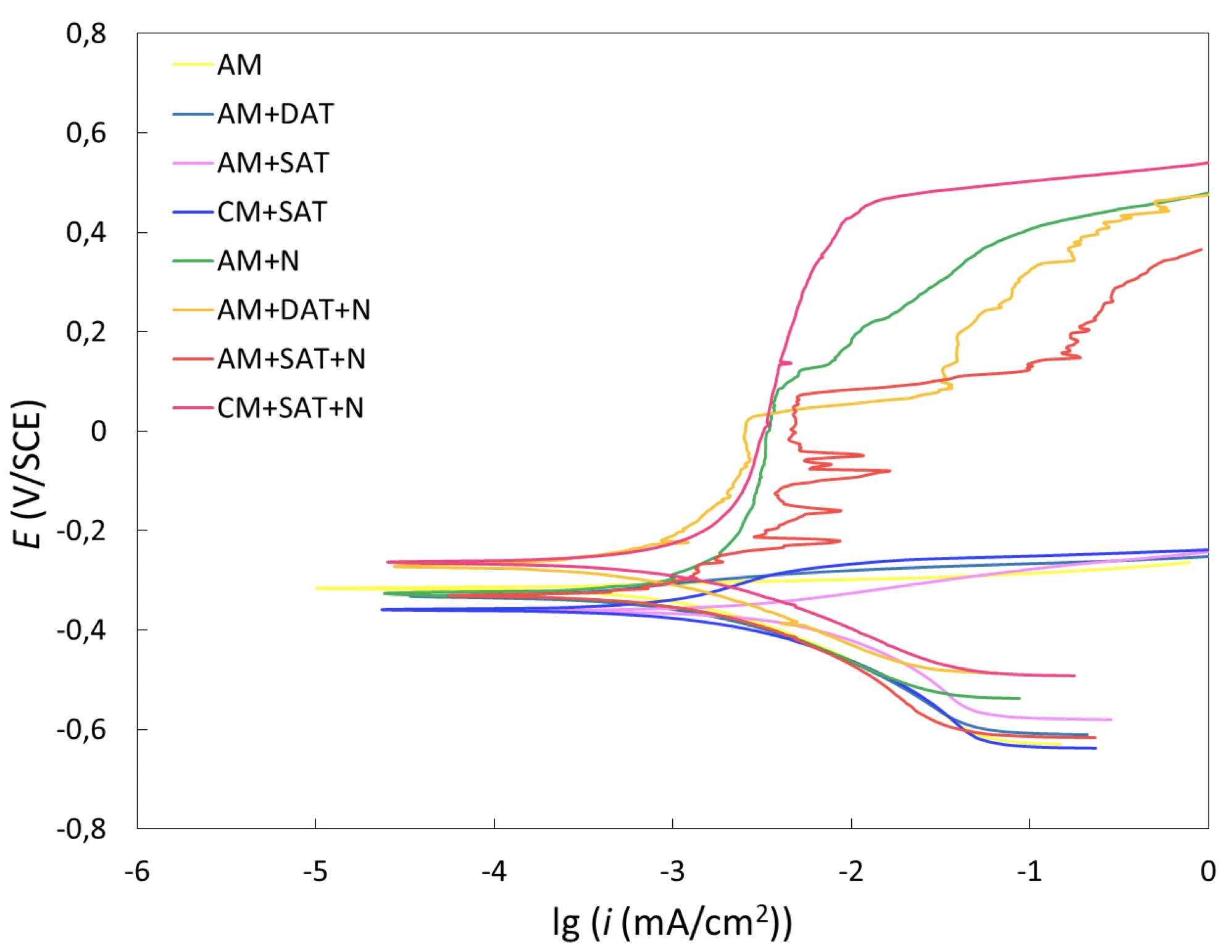


**Figure S4**. Potentiodynamic curves for AM and CM maraging steel after different heat treatments in 3.5 % NaCl.
